# Supplementary material for: Demonstration of hypergraph-state quantum information processing
Source: Nat Commun. 2024 Mar 23;15:2601. doi: 10.1038/s41467-024-46830-7 (PMC10960808; doi:10.1038/s41467-024-46830-7)
Supplement: Supplementary file 1 — Supplementary Information [file 41467_2024_46830_MOESM1_ESM.pdf]

# Supplementary Information: Demonstration of hypergraph-state quantum information processing


Jieshan Huang[1,6], Xudong Li[1,6,⋆], Xiaojiong Chen[1], Chonghao Zhai[1], Yun Zheng[1], Yulin Chi[1], Yan Li[1,2,3,4,5], Qiongyi He[1,2,3,4,5], Qihuang Gong[1,2,3,4,5], and Jianwei Wang[1,2,3,4,5,†]

[1] *State Key Laboratory for Mesoscopic Physics, School of Physics, Peking University, Beijing, 100871, China*
[2] *Frontiers Science Center for Nano-optoelectronics & Collaborative Innovation Center of Quantum Matter, Peking University, Beijing, 100871, China*
[3] *Collaborative Innovation Center of Extreme Optics, Shanxi University, Taiyuan 030006, Shanxi, China*
[4] *Peking University Yangtze Delta Institute of Optoelectronics, Nantong 226010, Jiangsu, China.*
[5] *Hefei National Laboratory, Hefei 230088, China*
[6] *These authors contributed equally to this work.*
⋆ *present address: John A. Paulson School of Engineering and Applied Sciences, Harvard University, Cambridge, MA, USA.*
† *emails to: jww@pku.edu.cn*


## Supplementary Note 1: Optimal projection bases for the Mermin inequalities measurements

As mentioned in the main text and Methods, the observables for the Mermin inequalities measurements are defined derivatively with the knowledge of local projectors $\{a_i, a'_i\}$ for each qubit, which can be obtained by a classical optimising algorithm to reach a maximal violation.

**Optimisation 1:** finding best measurement basis for Mermin inequalities

**Result:** Optimised basis set for all single qubits
**Input:** random initial measuring basis set $\{a_{ini}, a'_{ini}\}$, step size $\delta$, minimum step size $\delta_{min}$, maximum number of iteration $N$
**Output:** optimised basis set $\{a_{fin}, a'_{fin}\}$ and theoretic Mermin value $\mu$

```
n = 1;
{a_best, a'_best} = {a_ini, a'_ini};
μ_best = Calculate μ for basis {a_ini, a'_ini};
while δ ≥ δ_min do
    while n ≤ N do
        {a_new, a'_new} = Generate new random basis from old basis {a_best, a'_best} within step size δ;
        μ_new = Calculate μ for basis {a_new, a'_new};
        if μ_new > μ_best then
            {a_best, a'_best} = {a_new, a'_new};
            μ_best = μ_new;
            n = 1;
        end
        else
            n = n + 1;
        end
    end
    n = 1;
    δ = δ_0/2;
end
return {a_best, a'_best} and μ_best;
```

Here, we list all these single-qubit projectors for each state by the following manner. Since arbitrary zero-trace single-qubit operators can be expressed as $\hat{O} = o_1\hat{X} + o_2\hat{Y} + o_3\hat{Z}$ where $\hat{X}, \hat{Y}, \hat{Z}$ are Pauli operators, we do the same for $\{a_i, a'_i\}$ and use three components $\{x_i, y_i, z_i\}$ and $\{x'_i, y'_i, z'_i\}$ to denote each of them. Each component is given with an accuracy of 3 significant digits.

| | $q_1$ | $q_2$ | $q_3$ | $q_4$ |
|---|---|---|---|---|
| 1 | $(-1.76\times10^{-4}, 0.337, -0.941)$ | $(0.730, -0.683, -2.59\times10^{-5})$ | $(-0.276, -0.961, -1.73\times10^{-5})$ | $(-5.67\times10^{-5}, -0.276, 0.961)$ |
| | $(1.06\times10^{-4}, -0.730, -0.683)$ | $(-0.338, -0.941, 1.13\times10^{-4})$ | $(0.407, -0.913, -1.31\times10^{-4})$ | $(-2.98\times10^{-5}, -0.407, -0.913)$ |
| 2 | $(0.898, -0.439, 6.49\times10^{-5})$ | $(0.773, -0.634, 1.25495\times10^{-4})$ | $(0.106, -0.994, 7.44\times10^{-5})$ | $(0.584, -0.812, 7.47\times10^{-5})$ |
| | $(0.439, 0.898, -5.72\times10^{-5})$ | $(0.634, 0.773, 2.91\times10^{-5})$ | $(0.994, 0.106, 1.07\times10^{-4})$ | $(0.812, 0.584, -2.75\times10^{-5})$ |
| 3 | $(-0.514, 0.618, 0.595)$ | $(0.514, -0.617, -0.595)$ | $(0.514, -0.618, -0.595)$ | $(0.514, -0.618, -0.595)$ |
| | $(0.514, 0.617, -0.595)$ | $(-0.513, -0.618, 0.595)$ | $(-0.514, -0.617, 0.595)$ | $(-0.514, -0.618, 0.595)$ |
| 4 | $(0.985, 1.62\times10^{-4}, -0.172)$ | $(-0.136, -8.97\times10^{-5}, -0.991)$ | $(0.897, 1.40\times10^{-4}, 0.441)$ | $(-0.897, -8.42\times10^{-6}, -0.442)$ |
| | $(0.0870, -1.89\times10^{-5}, 0.996)$ | $(0.975, -7.16\times10^{-5}, -0.221)$ | $(0.940, -1.16\times10^{-4}, -0.340)$ | $(-0.940, -4.17\times10^{-5}, 0.340)$ |
| 5 | $(-0.456, -0.670, 0.586)$ | $(-0.448, -0.684, 0.576)$ | $(0.451, 0.678, -0.580)$ | $(-0.451, 0.678, 0.580)$ |
| | $(0.447, -0.685, -0.575)$ | $(0.455, -0.671, -0.585)$ | $(0.452, -0.676, -0.582)$ | $(0.452, 0.677, -0.581)$ |
| 6 | $(0.680, 0.681, 0.270)$ | $(0.142, 0.596, 0.790)$ | $(0.937, 0.292, 0.190)$ | $(0.119, -0.741, 0.661)$ |
| | $(0.678, -0.683, 0.270)$ | $(-0.110, 0.784, -0.611)$ | $(-0.938, 0.291, -0.190)$ | $(0.135, 0.648, 0.750)$ |

| | | | | |
|---|---|---|---|---|
| 7 | $(-0.0186, 0.804, 0.595)$ | $(0.684, -0.645, 0.341)$ | $(-0.674, -0.658, -0.335)$ | $(-0.0214, -0.701, 0.712)$ |
| | $(0.0229, 0.623, -0.782)$ | $(-0.667, -0.668, -0.331)$ | $(0.677, -0.655, 0.337)$ | $(0.0205, -0.737, -0.675)$ |
| 8 | $(-0.672, -0.699, 0.243)$ | $(0.244, -0.423, 0.872)$ | $(0.0524, 0.981, 0.186)$ | $(-0.00736, 1.00, -0.0283)$ |
| | $(0.699, -0.669, -0.253)$ | $(-0.117, -0.901, -0.417)$ | $(0.265, -0.188, 0.946)$ | $(0.269, 0.0285, 0.963)$ |
| 9 | $(0.959, 6.09 \times 10^{-5}, -0.283)$ | $(0.384, 2.07 \times 10^{-5}, 0.923)$ | $(0.999, -3.18 \times 10^{-5}, -0.0532)$ | $(0.965, -8.76 \times 10^{-5}, -0.261)$ |
| | $(0.333, 1.20 \times 10^{-4}, 0.943)$ | $(-0.942, -9.69 \times 10^{-5}, 0.336)$ | $(0.852, -2.51 \times 10^{-5}, 0.524)$ | $(0.311, -6.05 \times 10^{-5}, 0.950)$ |
| 10 | $(-0.374, 0.621, 0.689)$ | $(0.475, 8.83 \times 10^{-4}, -0.880)$ | $(-0.182, -0.924, 0.336)$ | $(-0.101, 0.977, 0.188)$ |
| | $(0.301, 0.774, -0.557)$ | $(6.80 \times 10^{-4}, -1.00, -0.00289)$ | $(-0.442, 0.374, 0.815)$ | $(0.465, 0.207, -0.861)$ |
| 11 | $(0.277, -0.128, -0.952)$ | $(0.810, -0.541, 0.228)$ | $(0.154, 0.826, -0.542)$ | $(-0.579, -0.812, 0.0734)$ |
| | $(-0.809, 0.541, -0.229)$ | $(0.275, -0.128, -0.953)$ | $(-0.985, -0.171, -0.0331)$ | $(-0.0727, -0.365, -0.928)$ |
| 12 | $(-0.0291, 0.583, -0.812)$ | $(-0.881, -0.456, -0.126)$ | $(0.492, -0.601, -0.630)$ | $(-0.881, -0.456, -0.126)$ |
| | $(-0.491, 0.594, 0.637)$ | $(0.465, -0.364, 0.807)$ | $(-0.0242, 0.576, -0.817)$ | $(0.465, -0.364, 0.807)$ |
| 13 | $(-0.124, 7.86 \times 10^{-5}, -0.992)$ | $(0.999, -3.07 \times 10^{-6}, -0.0369)$ | $(-0.115, -6.47 \times 10^{-5}, -0.993)$ | $(-0.125, -1.86 \times 10^{-5}, -0.992)$ |
| | $(-0.999, 9.53 \times 10^{-5}, 0.0459)$ | $(-0.115, -4.58 \times 10^{-5}, -0.993)$ | $(-0.999, 5.04 \times 10^{-5}, 0.0373)$ | $(-0.999, 7.92 \times 10^{-5}, 0.0469)$ |
| 14 | $(-0.591, -0.806, -1.11 \times 10^{-4})$ | $(-0.368, 0.660, 0.655)$ | $(0.369, -0.657, -0.658)$ | $(-1.28 \times 10^{-4}, -0.793, -0.609)$ |
| | $(0.806, -0.591, -3.01 \times 10^{-5})$ | $(-0.370, -0.655, 0.659)$ | $(0.369, 0.658, -0.657)$ | $(1.11 \times 10^{-4}, 0.609, -0.793)$ |
| 15 | $(0.315, 0.704, 0.637)$ | $(-0.776, 0.471, -0.420)$ | $(-0.313, -0.701, 0.641)$ | $(-0.636, 0.704, 0.315)$ |
| | $(0.313, 0.701, -0.641)$ | $(-0.776, 0.470, -0.420)$ | $(0.315, 0.704, 0.637)$ | $(0.642, 0.700, 0.313)$ |
| 16 | $(-0.663, -0.663, -0.346)$ | $(0.548, 0.548, 0.631)$ | $(0.419, 0.419, 0.805)$ | $(-0.601, -0.565, 0.565)$ |
| | $(-0.245, -0.245, 0.938)$ | $(0.447, 0.447, -0.775)$ | $(0.569, 0.570, -0.593)$ | $(-0.799, 0.426, -0.425)$ |
| 17 | $(-1.33 \times 10^{-5}, 0.997, 0.0770)$ | $(-5.20 \times 10^{-5}, -0.664, -0.748)$ | $(-0.628, -0.628, 0.460)$ | $(-0.629, 0.627, 0.460)$ |
| | $(9.65 \times 10^{-5}, -0.0771, 0.997)$ | $(-6.24 \times 10^{-5}, 0.748, -0.664)$ | $(0.628, -0.628, -0.460)$ | $(-0.627, -0.629, 0.459)$ |
| 18 | $(-0.776, -1.14 \times 10^{-4}, 0.631)$ | $(-0.980, -1.74 \times 10^{-4}, -0.199)$ | $(0.999, -2.16 \times 10^{-5}, 0.0534)$ | $(-0.398, 9.01 \times 10^{-7}, 0.917)$ |
| | $(0.631, 1.65 \times 10^{-4}, 0.776)$ | $(-0.199, 8.29 \times 10^{-5}, 0.980)$ | $(0.399, -7.87 \times 10^{-5}, -0.917)$ | $(0.999, 2.69 \times 10^{-5}, 0.0530)$ |
| 19 | $(-9.19 \times 10^{-5}, 0.873, 0.487)$ | $(1.00, -3.17 \times 10^{-5}, 1.60 \times 10^{-4})$ | $(0.577, 0.577, 0.577)$ | $(-0.730, -0.684, 9.39 \times 10^{-5})$ |
| | $(2.94 \times 10^{-6}, -0.487, 0.874)$ | $(1.00, 7.86 \times 10^{-6}, 1.67 \times 10^{-4})$ | $(0.577, -0.577, 0.577)$ | $(0.684, -0.729, -8.75 \times 10^{-5})$ |
| 20 | $(-0.598, 0.534, 0.598)$ | $(0.598, -0.534, -0.598)$ | $(-1.00, 1.43 \times 10^{-4}, 6.03 \times 10^{-5})$ | $(2.74 \times 10^{-5}, -0.598, -0.802)$ |
| | $(0.598, 0.535, -0.598)$ | $(-0.598, -0.535, 0.598)$ | $(-1.00, -4.10 \times 10^{-5}, -6.47 \times 10^{-5})$ | $(-8.85 \times 10^{-5}, -0.597, 0.802)$ |
| 21 | $(0.888, -0.460, -0.00478)$ | $(-0.888, -0.460, -0.00465)$ | $(-0.00586, -0.578, -0.816)$ | $(-0.00611, -0.578, 0.816)$ |
| | $(-0.888, -0.460, -0.00469)$ | $(-0.888, 0.460, 0.00489)$ | $(-0.00596, -0.577, 0.816)$ | $(0.00590, 0.577, 0.817)$ |
| 22 | $(-0.190, 0.963, 0.190)$ | $(-0.706, 0.0545, -0.706)$ | $(-1.44 \times 10^{-4}, -0.976, 0.218)$ | $(-1.91 \times 10^{-4}, 0.171, -0.985)$ |
| | $(0.681, 0.268, -0.681)$ | $(0.0386, 0.999, 0.0386)$ | $(-5.77 \times 10^{-5}, 0.218, 0.976)$ | $(8.69 \times 10^{-6}, -0.985, -0.171)$ |
| 23 | $(-0.577, 4.66 \times 10^{-5}, 0.816)$ | $(0.816, -6.52 \times 10^{-6}, 0.578)$ | $(0.816, -1.02 \times 10^{-4}, -0.577)$ | $(0.817, -1.28 \times 10^{-4}, 0.577)$ |
| | $(-0.577, 9.27 \times 10^{-6}, -0.817)$ | $(-0.817, -6.86 \times 10^{-5}, 0.577)$ | $(0.817, -1.26 \times 10^{-4}, 0.577)$ | $(-0.816, 7.23 \times 10^{-6}, 0.577)$ |
| 24 | $(-0.726, 0.562, 0.397)$ | $(-0.631, 0.450, 0.632)$ | $(0.629, 0.448, -0.636)$ | $(0.629, 0.448, -0.636)$ |
| | $(-0.726, -0.562, 0.397)$ | $(0.628, -0.448, 0.636)$ | $(0.630, 0.449, 0.633)$ | $(0.631, 0.449, 0.633)$ |
| 25 | $(4.10 \times 10^{-5}, -0.663, -0.748)$ | $(-0.325, -0.735, 0.595)$ | $(-0.345, -0.693, 0.633)$ | $(0.843, 0.538, -2.81 \times 10^{-5})$ |
| | $(5.66 \times 10^{-5}, 0.665, -0.747)$ | $(0.336, -0.713, -0.615)$ | $(0.315, -0.754, -0.577)$ | $(-0.843, 0.538, 1.65 \times 10^{-4})$ |
| 26 | $(-0.914, -6.22 \times 10^{-5}, 0.407)$ | $(-0.284, 0.659, -0.697)$ | $(0.283, 0.659, 0.696)$ | $(-0.595, 0.803, -2.26 \times 10^{-5})$ |
| | $(0.913, -1.26 \times 10^{-4}, -0.408)$ | $(0.278, 0.679, 0.679)$ | $(-0.278, 0.679, -0.679)$ | $(0.597, 0.802, 1.36 \times 10^{-4})$ |
| 27 | $(-0.494, -0.344, 0.799)$ | $(-0.152, -0.958, 0.246)$ | $(-0.444, 0.536, 0.718)$ | $(0.00103, 1.00, -0.00175)$ |
| | $(0.181, -0.939, -0.293)$ | $(0.503, -0.289, -0.814)$ | $(-0.282, -0.844, 0.456)$ | $(-0.526, 0.00188, 0.851)$ |
| 28 | $(0.914, 0.405, 1.15 \times 10^{-4})$ | $(-0.914, -0.405, 7.27 \times 10^{-5})$ | $(0.176, -0.794, -0.582)$ | $(0.176, 0.795, -0.581)$ |
| | $(-0.914, 0.406, -8.56 \times 10^{-5})$ | $(0.914, -0.406, -7.04 \times 10^{-5})$ | $(0.177, 0.794, -0.582)$ | $(-0.176, 0.793, 0.583)$ |
| 29 | $(-0.866, 1.23 \times 10^{-4}, -0.500)$ | $(-0.866, -1.32 \times 10^{-4}, 0.5000)$ | $(-0.866, -2.70 \times 10^{-5}, 0.500)$ | $(-0.866, 2.19 \times 10^{-5}, 0.500)$ |
| | $(0.866, 1.46 \times 10^{-4}, -0.500)$ | $(-0.866, 2.94 \times 10^{-5}, -0.500)$ | $(0.866, -1.25 \times 10^{-4}, 0.500)$ | $(0.866, -1.02 \times 10^{-4}, 0.500)$ |

## Supplementary Note 2: Analysis and discussion of the scalability of hypergraph state devices

In our scheme, the preparation of the multi-qubit hypergraph states relies on the realisation of multi-qubit controlled $C^m$-$Z$ gates. Implementing the $C^m$-$Z$ gates first requires the generation of $d$-dimensional (the local dimensionality of $d$) $n$-photon (the number of $n$ photons) entanglement, and then such quantum entanglement is translated from the photon sources to the entangling gates. In this case, the difficulty of realising the $C^m$-$Z$ gate is mapped to the generation of $d$-dimensional $n$-photon entangled states which have been recently realised[1,2]. Consequently, each qudit state is mapped to $\mathrm{Log}_2(d)$ qubits, among which arbitrary entangling gates can be applied deterministically. This allows the preparation of a number of $n\mathrm{Log}_2(d)$ qubits hypergraph states. In this section, we discuss the scalability of this scheme, which is represented by the photon count rate as an increase of the number of qubits.

The $d$-dimensional $n$-photon Greenberger-Horne-Zeilinger (GHZ) entangled states can be described as:

$$|\mathrm{GHZ}\rangle_{n,d} = \frac{1}{\sqrt{d}} \sum_{j=0}^{d-1} |j\rangle^{\otimes n}, \tag{1}$$

where $d$ denotes the local dimensionality and $n$ denotes the number of photons. Similar to the four-qubit hypergraph device as shown in Fig.2, which relies on the generation of two-ququart Bell entanglement states and the implementation of "space expansion – local unitary operation – coherent compression" process, we here apply the same process to the $(n-1)$ target photons, and we obtain the multi-qudit state as:

$$|\Phi\rangle(n,d) = \frac{1}{\sqrt{d^n}} \sum_{j=0}^{d-1} \sum_{k_1=0}^{d-1} \sum_{k_2=0}^{d-1} ... \sum_{k_{n-1}=0}^{d-1} |j\rangle_1 \otimes \hat{O}_{1,j}\hat{O}_{2,j}...\hat{O}_{n-1,j}|\phi_1\phi_2...\phi_{n-1}\rangle_2 \otimes |k_1k_2...k_{n-1}\rangle_3, \tag{2}$$

where $\hat{O}_{ij}(i = 1,2,...,n-1; j = 0,1,...,d-1)$ represents the $d$-dimensional local unitary operation applying on the $i$-th target qudit, $|j\rangle_1$ presents the control qudit, $|\phi_i\rangle_2$ presents the data qudit, and $|k_i\rangle_3$ presents the ancillary qudit, all in the logical basis. Performing the $d$-dimensional Hadamard gate on the qudit,

it returns a state as:

$$|\Phi\rangle'_H(n,d) = \frac{1}{\sqrt{d^n}} \sum_{j=0}^{d-1}\sum_{h_1=0}^{d-1}\sum_{h_2=0}^{d-1}...\sum_{h_{n-1}=0}^{d-1} |j\rangle_1 \otimes \hat{O}_{1,j}\hat{O}_{2,j}...\hat{O}_{n-1,j}|\phi_1\phi_2...\phi_{n-1}\rangle_2 \otimes |h_1h_2...h_{n-1}\rangle_3, \tag{3}$$

We rewrite the qudit 1 state in the d-dimensional Hadamard basis $|h_i\rangle (i = 0, 1, ..., n-1)$ as:

$$|\Phi\rangle'_H(n,d) = \sum_{k_1=0}^{d-1}\sum_{k_2=0}^{d-1}...\sum_{k_{n-1}=0}^{d-1} \frac{\sum_{i=0}^{d-1}|h_i\rangle_1 \otimes \sum_{j=0}^{d-1} h_{i,j}h_{j,k_1}h_{j,k_2}...h_{j,k_{n-1}}\hat{O}_{1,j}\hat{O}_{2,j}...\hat{O}_{n-1,j}|\phi_1\phi_2...\phi_{n-1}\rangle_2}{\sqrt{d^{n+1}}} \otimes |k_1k_2...k_{n-1}\rangle_3. \tag{4}$$

By projecting the auxiliary qudit into the superposition state corresponding to the controlled qudit state with correct phase compensations, this post-selection scheme results in the multiqudit multi-value controlled-unitary gate with a success probability of $1/d^3$, based on which we obtain the state as:

$$|\Phi\rangle'(n,d)_{\text{qudit}} = \frac{1}{\sqrt{d}}\sum_{j=0}^{d-1}|j\rangle \otimes \prod_{i=1}^{n-1}\hat{O}_{i,j}|\phi_i\rangle, \tag{5}$$

where subscript represents the encoding of information in the unit of qudits. We then apply the qudit-to-qubit mapping to realise the multi-qubit $C^m$-$Z$ gates. We encode the $\text{Log}_2(d)$-qubits information in one $d$-dimensional qudit. The qudit-encoding state in Eq.(5) can be rewritten as a $n\text{Log}_2(d)$-qubit-encoding state:

$$|\Phi\rangle'(n\text{Log}_2(d),2)_{\text{qubit}} = \frac{1}{\sqrt{d}}\sum_{j=0}^{d-1}\left(\prod_{s=0}^{\text{Log}_2(d)-1}|j_s\rangle\right) \otimes \prod_{i=1}^{n-1}\hat{O}_{i,j}\left(\prod_{t=0}^{\text{Log}_2(d)-1}|\phi_t\rangle_i\right). \tag{6}$$

where subscript represents the encoding of information in the unit of qubits, and $\forall j_s, \phi_t \in \{0,1\}$. In total, we have $m = n\text{Log}_2(d) - 1$ for the implementation of multi-qubit $C^m$-$Z$ gates. Importantly, the entangling operation between the $\text{Log}_2(d)$ qubits is deterministic using the $\hat{O}_{i,j}$. That results a success probability of $P_{\text{success}} = 1/d$ to implement the $C^m$-$Z$ gates, so as the preparation of the hypergraph states. Different hypergraph states can be realised by reconfiguring the $\hat{O}_{i,j}$ accordingly.

The counting rate of the hypergraph states is given by:

$$R_{\text{hypergraph}} = R_{\text{GHZ}} \cdot \alpha_{\text{loss}} \cdot P_{\text{success}}, \tag{7}$$

where $R_{\text{GHZ}}$ denotes the rate of generating the $|\text{GHZ}\rangle_{n,d}$ state, $\alpha_{\text{loss}}$ represents the loss of system, and $P_{\text{success}} = 1/d$ represents the success probability of the $C^m$-$Z$ gates. In order to estimate the maximal number of qubits in the generated hypergraph states, we choose state-of-the-art components and devices in the following calculations. The loss of system $\alpha_{\text{loss}}$ is given by $\alpha_{loss} = (\alpha_{\text{couple}} \cdot \alpha_{\text{SNSPD}})^n \cdot \alpha_{\text{circuit}}$, where the $\alpha_{\text{couple}}$ is the coupling efficiency from the quantum chip to fibers (we choose $\alpha_{\text{couple}} = 0.9$[4]), $\alpha_{\text{SNSPD}}$ is the efficiency of superconducting nanowire single-photon detectors (we choose $\alpha_{\text{SNSPD}} = 0.95$[5]), and $\alpha_{\text{circuit}}$ is the attenuation in the circuits composed of beamsplitters, crossers, and waveguides. The $R_{\text{GHZ}}$ can be estimated by:

$$R_{\text{GHZ}}(n,d) = R_0\eta^n_{\text{heralding}}p_0^{n/2}\frac{d}{d^n}, \tag{8}$$

where $R_0$ is the repetition rate of the pump, $\eta_{\text{heralding}}$ is the heralding efficiency of photon-pair sources, $p_0$ is the probability of generating one pair of photons, and $d/d^n$ is the probability of post-selecting the GHZ state from the $n^d$ states in the ideal case. The repetition rate $R_0$ is chosen as 500MHz, and the heralding efficiency $\eta_{\text{heralding}}$ is chosen as $\approx 0.9$ which is reported in an optimal silicon-based source[6]. The probability of generating photons $p_0$ in parametric sources is typically set as 5%-10% to avoid multiphoton terms within one source. Note that the deterministic single-photon sources[7] based on semiconductor quantum dots are not considered in this calculation, as it remains challenging to couple single photons into waveguides while maintaining high performance, and it requires further investigations to generate the $|\text{GHZ}\rangle_{n,d}$ states with a high successful probability. Moreover, the recent multiplexed heralding parametric source shows a factor of 9.7(5) enhancement in efficiency[8], i.e., $p_0 \approx 0.667$, which shows the possibility of implementing those multiplexed sources in the integrated photonic systems.

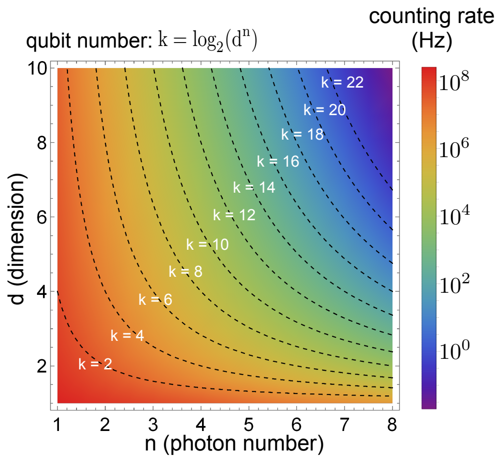


**Supplementary Figure 1 Estimation of photon counting rates for the multi-qubit hypergraph states.** In this estimation, we choose state-of-the-art quantum photonic components and devices: repetition rate $R_0 = 500$MHz, heralding efficiency $\eta_{\text{heralding}} = 0.9$, photon generation probability $p_0 = 0.667$, coupling efficiency $\alpha_{\text{couple}} = 0.9$, detection efficiency $\alpha_{\text{SNSPD}} = 0.95$, circuit transmission $\alpha_{\text{circuit}} = 0.8$, and successful probability $P_{sucess} = 1/d$. The counting rates are coded by colors and the key is provided at the right side. Contour lines (dashed) indicate the number of qubits corresponding to $n\text{Log}_2(d)$.

Supplementary Figure 1 shows the estimated counting rates of hypergraph states with a number of $n\text{Log}_2(d)$ qubits, when considering the above state-of-the-art quantum photonic components and devices. The contour lines (dashed) indicate the number of qubits corresponding to $n\text{Log}_2(d)$. We estimate that more than 20-qubit hypergraph states are achievable by combing those state-of-the-art quantum photonic devices and technologies. Further improvements

can be achieved by a full integration of near-deterministic single-photon sources, ultra-low-loss quantum circuits, and integrated high-efficiency single-photon detectors. Our scheme for hypergraph-state generation, relying on the entanglement translation and the qudit-to-qubit mapping, is a type of noisy intermediate-scale quantum (NISQ) device, which could play an enabling role in the near term to porotype the hypergraph-based quantum computing and information processing with only Pauli measurement that well suited for the blind version of MBQC. For example, in our current work, what we focused on is to benchmark the advantages of using hypergraph states for Pauli-universal MBQC. Certainly, other schemes/models capable of implementing a sequence of $C^m$-$Z$gates allow the preparations of hypergraph states and implementations of hypergraph-based computing. Eventually, it relies on the preparation of large-scale hypergraph states with the capability of performing error corrections. The universal schemes such as standard measurement-based quantum computing[9,10] and fusion-based quantum computing[11], in principle, could be adapted to implement the hypergraph-based MBQC with the capabilities of performing error corrections.

## Supplementary References


1. M. Erhard *et al.* Experimental Greenberger–Horne–Zeilinger entanglement beyond qubits. *Nature Photonics* **12,** 759–764 (2018).
2. J. Bao *et al.* Very-large-scale integrated quantum graph photonics. *Nature Photonics* **17,** 573–581 (2023).
3. Y. Chi, J. Huang & Z. Zhang. A programmable qudit-based quantum processor. *Nature Communications* **13,** 1166 (2022).
4. Y. Ding *et al.* Fully etched apodized grating coupler on the SOI platform with -0.58dB coupling efficiency. *Optics Letters* **39,** 5348–5350 (2014).
5. I. E. Zadeh *et al.* Superconducting nanowire single-photon detectors: A perspective on evolution, state-of-the-art, future developments, and applications. *Applied Physics Letters* **118,** 190502 (2021).
6. S. Paesani *et al.* Near-ideal spontaneous photon sources in silicon quantum photonics. *Nature Communications* **11,** 2520 (2020).
7. N. Tomm *et al.* A bright and fast source of coherent single photons. *Nature Nanotechnology* **16,** 399–403 (2021).
8. F. Kaneda & P. G. Kwiat. High-efficiency single-photon generation via large-scale active time multiplexing. *Science Advances* **5,** eaaw8586 (2019).
9. R. Raussendorf & H. J. Briegel. A one-way quantum computer. *Physical Review Letters* **86,** 5188–5191 (2001).
10. M. Gimeno-Segovia *et al.* From three-photon Greenberger-Horne-Zeilinger states to ballistic universal quantum computation. *Physical Review Letters* **115,** 020502 (2015).
11. S. Bartolucci *et al.* Fusion-based quantum computation. *Nature Communications* **14,** 912 (2023).
